# Cluster parameter-based DBSCAN maps for image characterization

**DOI:** 10.1016/j.csbj.2025.02.037

**Published:** 2025-02-28

**Authors:** Péter Bíró, Bálint Barna H. Kovács, Tibor Novák, Miklós Erdélyi

**Affiliations:** Department of Optics and Quantum Electronics, University of Szeged, Dóm tér 9, Szeged, 6720, Hungary

**Keywords:** SMLM, Cluster analysis, DBSCAN, Image characterization, Parameter optimization

## Abstract

Single-molecule localization microscopy techniques are one of the most powerful methods in biological studies, allowing the visualization of nanoclusters. Cluster analysis algorithms are used for quantitative evaluation, with DBSCAN being one of the most widely used. Clustering results are extremely sensitive to the initial parameters; thus, several methods including DBSCAN maps, have been developed for parameter optimization. Here, we introduce cluster parameter-based DBSCAN maps, which are directly applicable to measured datasets. These maps can be used for image characterization and parameter optimization through sensitivity studies. We show the applicability of these maps to simulated and measured datasets and compare our results with the recently implemented lacunarity analysis for SMLM measurements.

## Graphical abstract

**Figure:**
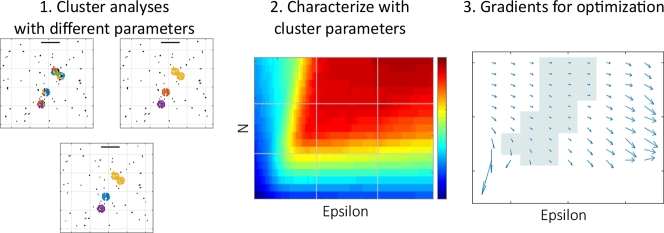

## 1 Introduction

The nanoscale organization of proteins and biomolecules into complex structures is one of the most critical and widely investigated areas of cell biology [1], [2], [3], [4]. However, the direct visualization of such structures is challenging because of their small size, low-contrast detectability, and the sensitivity of the local environment. Optical superresolution techniques have paved the way for such investigations. Single-molecule localization microscopy (SMLM) techniques such as (fluorescent) photoactivation localization microscopy [5], [6], (direct) stochastic optical reconstruction microscopy [7], [8], points accumulation for imaging in nanoscale topography [9], ground state depletion microscopy followed by individual molecule return [10] and minimal photon fluxes [11] provide subdiffraction spatial resolution enabling the study of cellular structures [12], [13], [14]. The localization data generated by SMLM methods come in the form of a point cloud, represented as a list of coordinates of the localized emitters. Novel analytical approaches are required for such pointillistic datasets, which differ from conventional pixelated images. This data format makes them amenable to statistical methods for describing spatial arrangement [15], [16], [17], in particular cluster analysis [18], [19]. Different global clustering methods, which return an ensemble result, have been applied to SMLM measurements, including Ripley's functions [20], [21], [22], nearest neighbor analysis [23], [24], pair correlation [25], [26], and lacunarity [27], [28]. These methods can be used to determine if the protein distribution is complete spatial randomness or clustered [29], and can be combined with persistent homology and principal component analysis [30]. However, complete clustering methods (which classify all localizations) provide a more detailed description of the data and are increasingly popular for SMLM analysis. These methods are typically based on density, e.g., density-based spatial clustering of applications with noise (DBSCAN) [31], tessellation [32], [33], Bayesian approaches [34], [35], or machine learning [36]. Recent clustering techniques include the localization precision [37], use grid-based clustering [38], combine different methods like intensity-based segmentation and Voronoi tessellation [39], and extend existing methods to 3D datasets [40], [41]. DBSCAN remains one of the most popular clustering methods [42], [43], [44], [45], [46] because it is adaptable to diverse clustering conditions (as it requires more user inputs) and robust to multiple blinking [47]. The DBSCAN algorithm requires two parameters: the minimum number (N) of points that form a cluster and the maximum distance (epsilon) between adjacent points. However, the results of the DBSCAN analysis are extremely sensitive to initial parameters. Several methods have been applied to optimize the parameters of the clustering algorithm [48], [49], [50], as well as the parameter ranges [51]. Different DBSCAN map methods have also been used for optimization, e.g., comparing a measurement with different simulated datasets (where the ground truth and hence the ideal parameters are known) [47], and comparing the clustering results at the localization level for different parameters (close to the ideal values, small variations should give similar clustering results) [52].

Here, we study the use of cluster parameter-based DBSCAN maps for image characterization, sensitivity studies, and DBSCAN parameter optimization. These maps can be calculated without knowing the ground truth and are hence directly applicable to measured datasets. They rely only on the cluster properties and not on the individual localizations that form these clusters. The maps themselves can be used as a global clustering method, but based on the map gradients, an ideal range of parameters can be determined and used as a complete clustering method. The optimal parameters depend on the desired clustering outputs (e.g., clusters given by individual target molecules – the response function –, or clustered target molecules), which appear in different map regions.

## 2 Methods

### 2.1 Cluster parameter-based DBSCAN maps

DBSCAN maps were generated for both simulated and measured datasets. Cluster analysis was performed with different (epsilon, N) parameter pairs, and cluster parameters (e.g., the cluster area defined by the convex hull of the localizations, and the localization number) were saved for all pairs. When evaluating the measurements, epsilon was varied from 10 to 90nm, a domain comparable with the size of the examined nanostructures. N was varied from 2 to 20. The localization precision limits the epsilon resolution of these maps; hence we used a step size of 10nm for epsilon and 2 for N. Simulated datasets contain more precise and much fewer localizations; therefore, a step size of 1nm and 1 was used for epsilon and N, respectively. Based on the saved cluster parameters, five maps were generated for all the different simulation parameters and measurement treatments, i.e. average (AV) cluster area and localization number, standard deviations (SD), and cluster number [35]. All maps display results on a logarithmic scale to highlight changes.

### 2.2 TestSTORM simulations

**Fig. 1:**
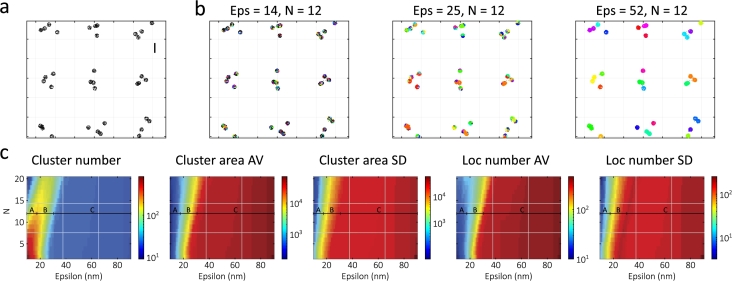
(a) Example of a simulated structure, scale bar: 500 nm; (b) DBSCAN analysis result for three different input parameter pairs ((14 nm, 12), (25 nm, 12), and (52 nm, 12)) from each regime; (c) the five DBSCAN maps that characterize the sample showing three different regimes.

Simulated datasets were generated using the TestSTORM simulation software [53]. We used the inbuilt “disk pattern” generator to create nine circular disks in a 3×3 grid (with a spacing length of 2500nm) consisting of several nanofoci distributed randomly within the circular areas (Fig. 1a), as described in previous work [28]. The cluster radius was set to 560nm, with a nanofocus density of $4\;\mathrm{nanofoci}/\mu m^{2}$ containing 150 labels per nanofocus. Image stacks containing 8,000 frames were generated with different linker lengths (the nanofocus size) and nonspecific localization densities (the background). Table 1 displays the pattern and labeling parameters. The dye (AF647) and acquisition parameters were set to default values (Supplementary Table S1). Generated image stacks were evaluated (localization and filtering) using the rainSTORM reconstruction software [54].

**Table 1:** Pattern and labeling parameters used in the simulations.

	Nanofocus size	Background
Radius (nm)	560	560
Dens (1/μm^2^)	4	4
Mean N of labels/nanofocus	150	150
Var. N of labels/nanofocus	5	5
Length of linkers (nm)	55-99-143	99
Non-spec. l. dens. (1/μm^3^)	0	0-10-30

### 2.3 DNA DSB dSTORM measured images

DNA double-strand breaks (DSBs) can produce highly pathological genomic instabilities that, if left un-repaired, can lead to cell death or drive the development of cancer [55], [56], [57]. The repair mechanisms are executed by proteins that accumulate around the DSBs. The phosphorylation of H2AX, forming yH2AX, is a highly specific and sensitive molecular marker of DNA damage and repair [58], [59]. To demonstrate the effectiveness of our technique, we used dSTORM datasets from a previous study where radiation-induced DNA DSBs were visualized using AlexaFluor 647-labeled yH2AX molecules [60]. The kinetics of the DSBs can be modelled as follows: the number of DSBs created by the irradiation is proportional to the radiation dose, reaches a maximum around 30 min post irradiation, and follows an exponential decay function over time as they are getting repaired [61], [62], [63]. As the total dose increases, DSBs may co-localize within individual radiation-induced foci, and therefore the cluster area and the number of localizations within a cluster (which is proportional to the number of target proteins) are both relevant parameters to characterize the foci [64].

### 2.4 Comparison with lacunarity

We compared our results with those of the lacunarity global clustering method, which was recently implemented for SMLM datasets [28]. In summary, this method calculates the box masses (the number of localizations inside the box) for different box positions; lacunarity is then calculated from the sums of the first and second moments of the box masses. A lacunarity curve is determined for both the sample and for a random dataset with the same number of localizations, using different box sizes. The relative difference of the two lacunarity values for each box size gives the lacunarity difference curve (LDC) of the sample, which characterizes its heterogeneity on different scales. Box sizes were varied from 1nm to 10μm, and changes in the peak position of the LDC curve indicate the scale at which the changes occur.

A quantitative analysis often uses the lacunarity value for a given box size to compare different samples [65], [66]. This is analogous to performing a DBSCAN analysis on a given pair (epsilon, N) of values. The evaluation of the whole lacunarity curve (hence the peak position of the LDC) is analogous to using the DBSCAN maps, which can provide information on different scales, and can determine the input parameters for the quantitative analysis. Power laws are a form of scale invariance that have been observed in several scientific fields [67], [68]. While scale invariance can be challenging, the regions where the image shows fractal (self-similar) properties can be derived from the lacunarity curve by plotting on a log-log scale [69]. Similarly, scale-invariant regions can be obtained from the DBSCAN map data, as cluster parameters can be plotted against epsilon on a log-log scale.

## 3 Results

### 3.1 TestSTORM

#### 3.1.1 Introducing maps through an example

To demonstrate the applicability of the cluster parameter-based DBSCAN maps, we examined their characteristics and the clustering results in different regions. We selected the simulation dataset with a linker length of 99nm and zero background. The calculated maps and three clustering results are displayed in Figs. 1b and 1c.

These maps display three different regimes. In regime A, the cluster number increases with epsilon as cluster cores form. Here, the AV values have a weak epsilon dependence, but the SD values also increase with epsilon as the cores grow and other cores appear. In regime B, as epsilon increases further, cluster cores start to merge and the cluster number decreases. The AV values increase rapidly, as the merged cores become significantly larger and contain more localizations. The SD values also increase, reaching a local maximum at the edge of the regime. Finally, in regime C, all five parameters settle to near constant values as the algorithm finds different nanofoci. Here, the SD values have a local minimum, as the nanoclusters have similar properties. Both the AV and SD values appear to increase as neighboring nanoclusters merge.

The five parameters that characterize the clustered image depend strongly on the input parameters, even within a given sample. To investigate the sample dependence of these maps, we examined two sample parameters: the nanofocus size and the density of background localizations.

#### 3.1.2 Nanofocus size dependence

**Fig. 2:**
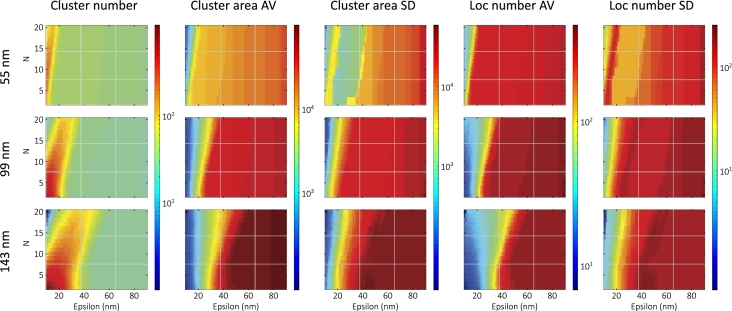
Calculated DBSCAN maps (with the same color bars) for different nanofocus sizes (55, 99, and 143 nm).

To achieve different nanofocus sizes, we changed the length of linker parameter in TestSTORM (as the labels are placed around the point-like epitopes over a spherical shell). The calculated maps for the three different linker lengths (55, 99, and 143nm) are shown in Fig. 2.

In the case of the smallest nanofocus size (55nm), cluster cores grow and merge more rapidly as a function of epsilon, compared to the previous (99nm) case. Hence, the first two regimes are narrowed and all five parameters show a strong epsilon dependence. This narrowing can be quantified by fitting the edge of the cluster area AV maps (Supplementary Figure S3): the epsilon axis intercept moves from 20.40nm to 10.60nm. Small nanofoci are better separated, resulting in smaller AV and SD values in the third regime, and slightly higher cluster number values. However, neighboring nanofoci still merge at higher epsilon values, producing discontinuous steps in the AV and SD maps. Increasing the nanofocus size to 143nm causes the first two regimes to broaden and the initial epsilon dependence to weaken. The epsilon axis intercept moves from 20.40nm to 32.57nm in this case (Supplementary Figure S3). Nanoclusters are separated at higher epsilon values as their labeling density decreases. However, more nanofoci overlap, resulting in cluster sizes exceeding that of the average nanofocus, and increasing both the AV and SD values.

Lacunarity findings show that changes in the nanofocus size have a greater effect on smaller box sizes, moving the LDC peak towards the larger box sizes (30nm → 88nm) [28]. These results are consistent with our findings, showing a greater effect on the initial epsilon dependence (smaller epsilon values).

#### 3.1.3 Background dependence

**Fig. 3:**
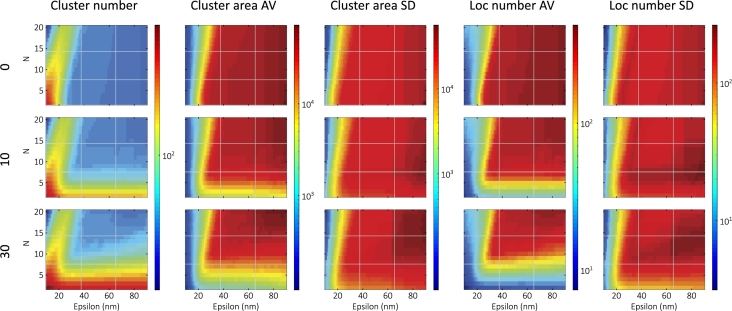
Calculated DBSCAN maps (with the same color bars) for different nonspecific label densities (0, 10, and $30\;\mu m^{-3}$).

Nonspecific binding and background signals have been an ongoing challenge in SMLM. Various techniques have been applied to reduce them [70], [71]. However, in cluster analysis, nonclustered target molecules also contribute to the background. To examine the effect of such localizations, we added nonspecific labels to the previous 99nm linker length simulation (the middle value), with a density of 10 and $30\;\mu m^{-3}$. The calculated maps are shown in Fig. 3.

In the case of nonspecific localizations, the maps display three effects. First, the cluster number increases significantly in the low-epsilon (<20nm), low-N (<5) region. In this area, characterized by the localization precision, the other maps do not change significantly, indicating the appearance of new cluster cores with similar properties. Second, with increasing epsilon values (>20nm), the maps change only in the low-N (<10) regions. Background localizations can form small clusters containing close to the minimum number of points. This suggests that clusters formed by localizations generated by single target molecules (the response function) are found within this band of low-N values. These clusters decrease the average area and localization number significantly. For extremely low-N values, where most clusters are small, the SD values fall below the zero-background values. However, as N increases and these clusters begin to disappear, their number becomes comparable to the original clusters, and the SD values also increase. Third, the size of the affected band and the number of these small clusters depend on the nonspecific localization density. At a higher density, the effect of background localizations increases slightly towards the higher values of epsilon. This effect can be quantified by fitting the edge of the localization number AV maps (Supplementary Figure S4). The fitted slopes are $0.004\;nm^{-1}$ and $0.070\;nm^{-1}$ at nonspecific label densities of $10\;\mu m^{-3}$ and $30\;\mu m^{-3}$, respectively. As the density is further increased ($50\;\mu m^{-3}$), the fitted slope increases to $0.129\;nm^{-1}$ (data not shown, see Supplementary Figure S4).

Lacunarity findings show that increasing the nonspecific label density evenly homogenizes the whole image, and the LDC peak moves very slightly towards the smaller box sizes (69nm → 64nm), indicating a greater effect on larger box sizes [28]. This is also consistent with our results, which initially show a uniform change on the maps, increasing slightly towards the higher epsilon values at higher densities.

Many other simulation parameters could be examined, such as the number of clusters, the cluster area, or the number of labels per nanofocus, whose relative importance may depend on the problem of interest. However, because the main parameter used by the clustering algorithm is the localization density, these parameters are not completely independent. The effect of higher label numbers is similar to that of lower nanofocus sizes (when the other parameters are bound), as the labeling density is greater in both cases. An increase in cluster number or cluster size (for a given nanofocus density) also increases the total number of localizations and hence their density, which would also change the epsilon dependence of the maps.

### 3.2 Measurements

To test our method on biological measurements, we selected two dSTORM datasets from a previous study on the DSB repair mechanism [60]. The first dataset was used to investigate the effects of different dose levels of X-ray radiation in cell nuclei (dose-dependent measurements); the second was used to study the time evolution of DSB foci after irradiation (time-dependent measurements).

#### 3.2.1 Dose-dependent measurements

**Fig. 4:**
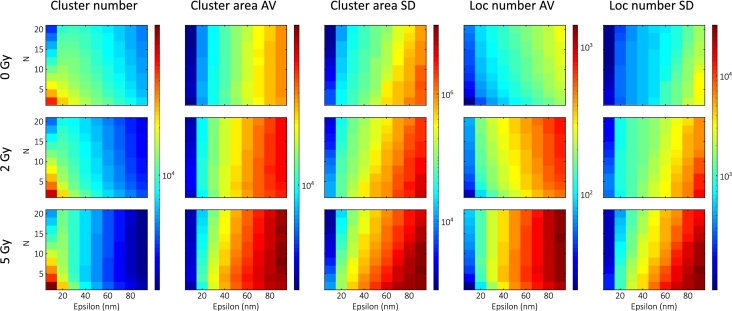
Calculated DBSCAN maps (with the same color bars) corresponding to different dose levels (0 Gy, 2 Gy, and 5 Gy).

For different dose levels of X-ray radiation, we selected cell groups that were either untreated (0Gy) or irradiated with 2Gy or 5Gy and fixed 30 minutes after irradiation. Higher irradiation doses produced more DSBs, which increased their density inside the cell nuclei. The maps corresponding to different dose levels are shown in Fig. 4.

For the untreated cells, the algorithm identified small clusters containing relatively few localizations. Compared to the control group, the cluster area and the localization number increased following the 2Gy treatment, as expected. However, following the 5Gy treatment, they increased further, together with the SD values. This behavior could result from the merging of different clusters due to the high cluster density. The rapid decrease in the number of clusters is also consistent with this result.

Compared with lacunarity, the peak of the LDC moves towards the smaller box sizes (49nm → 39nm), indicating that the change occurs on the scale of the foci and not of the nanofoci [28]. This is also consistent with our findings, showing that the average values do not change rapidly for small epsilon values (<20nm), as seen in the simulations.

#### 3.2.2 Time-dependent measurements

**Fig. 5:**
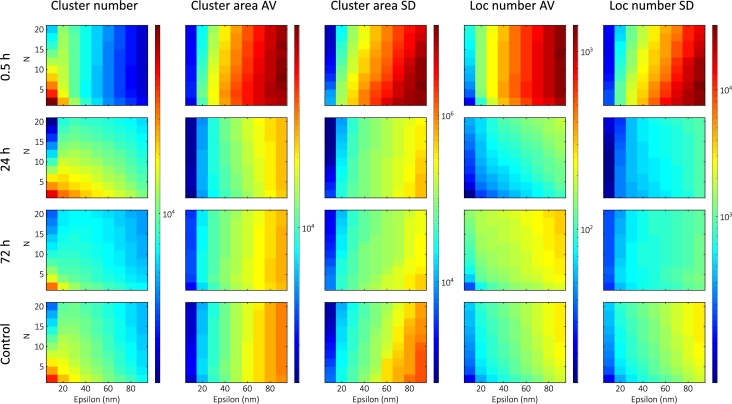
Calculated DBSCAN maps (with the same color bars) corresponding to different time intervals (30 min, 24 h, 72 h, and control).

Time-dependent studies were performed on cell groups that were fixed 30 minutes, 24 hours, or 72 hours after 5Gy irradiation. The cells fixed after 30 minutes were the same as in the previous section. The calculated maps for time-dependency are shown in Fig. 5.

**Fig. 6:**
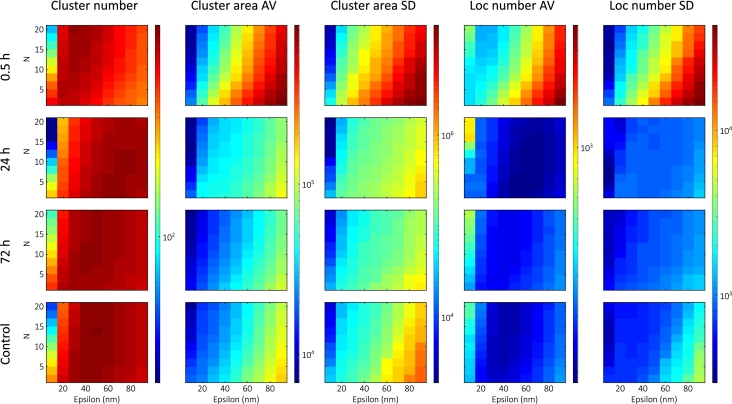
Calculated DBSCAN maps after filtering out the small clusters (with the same color bars) corresponding to different time intervals (30 min, 24 h, 72 h, and control).

As the DSBs undergo repair, the cluster density decreases, resulting in a drop in the average cluster size. The 24h group typically had the most clusters with the lowest number of localizations, in seeming contradiction to the assumption that the number of DSBs decreases over time. However, the simulations suggest that this can arise from an increase in the background localizations. To retain only the DNA damage-induced larger repair foci, we filtered out clusters smaller than $5000\;nm^{2}$ (approximately 80nm in diameter) given by the yH2AX background [72]. The filtered maps are shown in Fig. 6.

After filtering out the small clusters, the average cluster area follows the predicted decrease over time. Filtering lowers the number of clusters in the low-epsilon, low-N region significantly, thereby homogenizing the cluster number maps. There is also a significant change in the nature of the maps in the small-epsilon, high-N region for the 30min group.

The lacunarity results show that the LDC peak returns to the control value and that heterogeneity increases after repair [28]. Our finding shows that the average cluster values move towards the control values, and that the need for filtering stems from the change in heterogeneity.

#### 3.2.3 Process windows

**Fig. 7:**
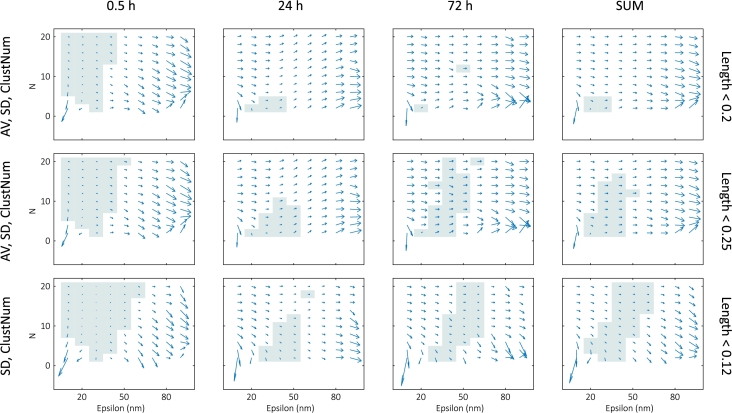
Sum gradient maps of the time-dependent measurements with process windows (for the SUM – the sum of the three different time groups – the length limit is also multiplied by 3).

Although samples within a given group generally show unignorable variations, these variations are smaller than those between the groups. Quantitative analysis can therefore be conducted in terms of the treatments applied to the different groups. Slight changes in the input parameters should not affect the feature of the final clustered image and should return similar outputs. This cluster variability with respect to the variation of the input parameters can be used to find unbiased clustering parameters [52]. Similar information can be obtained from these maps, as the gradient of the cluster parameter-based maps shows the sensitivity of the clustering output to the input parameters. To identify regions where the clustering algorithm output is less dependent on the input parameters, we calculated the sum gradient maps of the time-dependent measurements, shown in Fig. 7. For each cluster parameter, the gradients and thus the ideal regions could be determined. However, our aim was to optimize several parameters simultaneously. To achieve this, we normalized each DBSCAN map, calculated the gradients, and summed either all five maps (two AV, two SD, and the cluster number) or just three maps (two SD and the cluster number) for different treatments. The ideal region was determined by considering the length of the sum gradient (i.e., where it was smaller than an arbitrary threshold value). The sum of the sum gradients for different treatments could also be calculated (with an adjusted threshold), to optimize over the entire dataset (i.e., for all treatments).

The map values for the 24h and 72h groups are lower than for the 30min group, and therefore the gradients are also expected to be lower. However, since the maps are normalized first, the relative changes are higher, resulting in much smaller windows for these groups. This could be compensated by increasing the threshold on the sum gradient maps. Alternatively, the average maps could be omitted (creating the sum gradient maps based on the two SD and the cluster number maps), which would give a similar result. The different areas in these regions imply different clustering results: finding smaller clusters (background yH2AX, response function at lower values of epsilon and N) or finding larger clusters (DNA damage-induced repair foci at higher values of epsilon and N). Although some maps may suggest an ideal pair with lower epsilon and higher N (e.g., the 30min group), this region could be highly dependent on filtering, as seen above. These values should therefore usually be avoided. The two sets of parameters (epsilon, N) used in the previous work [60] are (25nm, 5) for smaller clusters and (50nm, 8) for larger clusters, consistent with our findings.

#### 3.2.4 Clustering outputs and biological phenomena

Using the parameter pair (50nm, 8) for quantitative evaluation, dose-dependent measurements (0, 2, and 5Gy) show a linear trend in the cluster area with increasing dose (0.041, 0.103 and $0.169\;\mu m^{2}$, respectively). The number of localizations also increases with the dose (218, 562, 750, respectively), indicating a higher number of proteins in the repair foci, although the trend is not exactly linear. Time-dependent measurements (30min, 24h, and 72h) show a rapid decrease in cluster area (0.169, 0.046, and $0.031\;\mu m^{2}$, respectively), with some overcompensation after 72 hours compared to control measurements. However, the number of localizations remains comparable (750, 196, 248), suggesting that the repair mechanisms are not fully completed (or that some irreversible changes have occurred after the 5Gy irradiation).

Moving towards the inside of the process window using the (40nm, 10) parameter pair, the dose-dependent cluster area values change to 0.030, 0.077 and $0.106\;\mu m^{2}$, respectively (average relative difference is 36.64%). It shows a similar trend to the (50nm, 8) values, but the number of localizations shows some saturation (208, 525, 594), indicating a limit to the total number of yH2AX in the cell nuclei. Time-dependent measurements show a slightly slower repair mechanism, but a similar overcompensation (0.106, 0.042 and $0.025\;\mu m^{2}$ respectively, with 243 localizations after 72 hours). The average of the relative differences of the examined cluster parameters is 17.91%.

Moving outside of the process window using the (60nm, 6) parameter pair, the dose-dependent cluster area trend is completely linear (0.062, 0.165 and $0.311\;\mu m^{2}$, average relative difference is 66.13%), with a similar trend in the localization number (244, 685, 1063). Time-dependent measurements show an extremely fast repair mechanism, where the 24h value is already under the control value (0.311, 0.056 and $0.044\;\mu m^{2}$). However, the localization number values at 24h (187) and 72h (272) are quite similar to the (50nm, 8) values (196, 248, respectively). The average of the relative differences of the examined cluster parameters is 28.11%. Supplementary Table S2 displays all cluster parameters for the three input parameter pairs.

## 4 Conclusion

In conclusion, we have shown that DBSCAN maps based only on cluster parameters can characterize an image to constitute a global clustering method. Simulation and measurement results yielded conclusions consistent with those of the lacunarity method. Calculating these maps can be time-consuming compared to the lacunarity analysis, especially if the correct method is used for the given dataset [73]. However, the sensitivity of these maps can be used to optimize the input parameters, and a complete clustering result can be obtained for further quantitative analysis.

## Code availability

A software (running in Matlab) to generate cluster parameter-based DBSCAN maps is available at https://titan.physx.u-szeged.hu/~adoptim/?page_id=1922.

## CRediT authorship contribution statement

**Péter Bíró:** Writing – original draft, Visualization, Software, Methodology, Investigation, Conceptualization. **Bálint Barna H. Kovács:** Methodology, Investigation. **Tibor Novák:** Writing – review & editing, Software. **Miklós Erdélyi:** Writing – review & editing, Supervision, Resources, Funding acquisition.

## Declaration of Competing Interest

The authors declare that they have no known competing financial interests or personal relationships that could have appeared to influence the work reported in this paper.

## Appendix A Supplementary material

MMC The Supplementary material includes additional testSTORM parameters, DBSCAN maps with logarithmic scales, quantitative parameters to characterize maps, and clustering results using different input parameters. MMC
