# Supplementary material for: Cluster parameter-based DBSCAN maps for image characterization
Source: Comput Struct Biotechnol J. 2025 Feb 28;27:920–7. doi: 10.1016/j.csbj.2025.02.037 (PMC11930167; doi:10.1016/j.csbj.2025.02.037)
Supplement: MMC — The Supplementary material includes additional testSTORM parameters, DBSCAN maps with logarithmic scales, quantitative parameters to characterize maps, and clustering results using different input parameters. [file mmc1.docx]

Supplementary information for „Cluster parameter-based DBSCAN maps for image characterization”

P. Bíró^a^, B. B. H. Kovács^a^, T. Novák^a^, M. Erdélyi^a^

^a^ Department of Optics and Quantum Electronics, University of Szeged, Dóm tér 9, Szeged, 6720, Hungary

**TestSTORM simulations**

| Dye parameters | | Acquisition parameters | |
| --- | --- | --- | --- |
| Emission WL (nm) | 665 | Frame rate (1/s) | 20 |
| Char. ON time (s) | 0.05 | Exp. Time (s) | 0.05 |
| Char. OFF time (s) | 420 | Pixel size (nm) | 160 |
| Bleaching constant (s) | 999999 | Av. BG level | 200 |
| Emitted photon/sec | 104000 | Struct. BG strength | [0 0] |
| Mean bonding angle (°) | 0 | RI of immersion m. | 1.518 |
| SD of bonding angle (°) | 360 | RI of sample m. | 1.331 |
|  |  | Numerical aperture | 1.4 |
|  |  | Electrons/count | 21.5 |
|  |  | Pre-amplification | 2.5 |
|  |  | Actual EM gain | 90 |
|  |  | Quantum efficiency | 0.9 |

Table S1: Dye and acquisition parameters used in the simulations.

**Quantitative outputs of cluster analyses**

|  |  | (50,8) | (40,10) | $\left\vert\delta_{rel} \right\vert$ (%) | (60,6) | $\left\vert\delta_{rel} \right\vert$ (%) |
| --- | --- | --- | --- | --- | --- | --- |
| 0 Gy 30 min | Area AV | 4.05·10^4^ | 2.99·10^4^ | 30.84 | 6.20·10^4^ | 53.05 |
|  | LocNum | 218 | 208 | 5.81 | 244 | 11.91 |
|  | ClustNum | 5688 | 5614 | 1.32 | 5284 | 7.10 |
| 2 Gy 30 min | Area AV | 1.03·10^5^ | 7.65·10^4^ | 33.55 | 1.65·10^5^ | 60.94 |
|  | LocNum | 562 | 525 | 10.70 | 685 | 21.86 |
|  | ClustNum | 3766 | 3921 | 8.13 | 3148 | 16.41 |
| 5 Gy 30 min | Area AV | 1.69·10^5^ | 1.06·10^5^ | 45.52 | 3.11·10^5^ | 84.40 |
|  | LocNum | 750 | 594 | 25.50 | 1063 | 41.87 |
|  | ClustNum | 3387 | 4188 | 30.47 | 2417 | 28.64 |
| 5 Gy 24 h | Area AV | 4.64·10^4^ | 4.16·10^4^ | 13.78 | 5.56·10^4^ | 19.74 |
|  | LocNum | 196 | 216 | 12.92 | 187 | 4.61 |
|  | ClustNum | 4679 | 3846 | 21.44 | 5283 | 12.91 |
| 5 Gy 72 h | Area AV | 3.09·10^4^ | 2.46·10^4^ | 25.49 | 4.36·10^4^ | 41.10 |
|  | LocNum | 248 | 243 | 3.01 | 272 | 9.75 |
|  | ClustNum | 5298 | 5264 | 0.25 | 4912 | 7.29 |

Table S2: Clustering outputs and relative differences (compared to the (50,8) values) with different input parameters.

**Logarithmic scale**

| 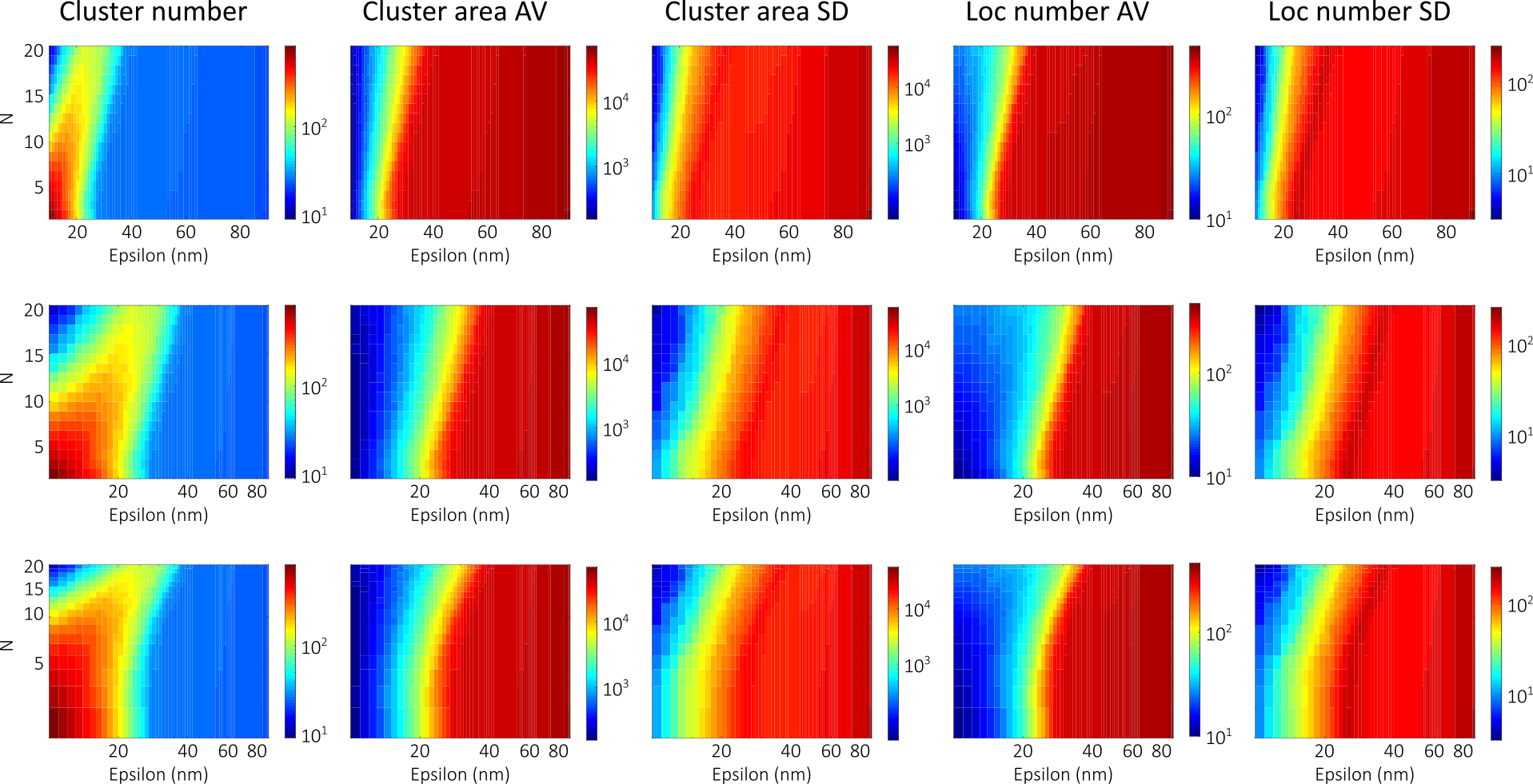 |
| --- |
| *Figure S1: DBSCAN maps from Figure 1 (first row) with logarithmic epsilon scale (middle row) and logarithmic epsilon and N scale (last row)* |

Logarithmic scales can highlight changes, such as the epsilon dependence of the average cluster area or the breaking point of the average localization number. However, the changes depend on the problem at hand (on the sample, or on the parameter range), and changing the scale is only a visualization: the values are the same, and a quantitative evaluation may be necessary to derive information from the images.

**Quantitative parameters**

Different parameters can be used to characterize the maps. For example, the slope of the ridge in the cluster number maps for different nanofocus sizes (Figure S2). The fitted slopes are 2.72, 1.17, and 0.64 nm^-1^, respectively.

| 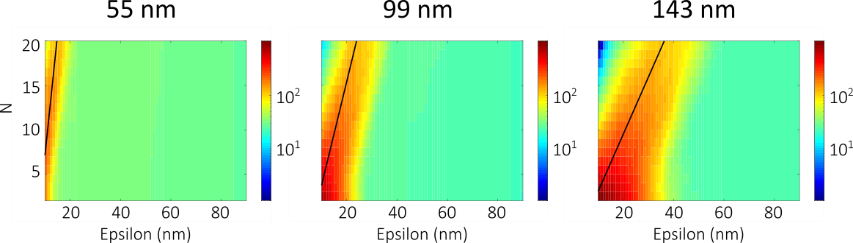 |
| --- |
| *Figure S2: Tilt fitting in the cluster number maps for different nanofocus sizes (55, 99, and 143 nm)* |

The edge of the cluster area AV maps (for each N, the epsilon position where the derivative of the area with respect to epsilon has its maximum value) carries similar information (Figure S3). The fitted slopes are 2.03, 1.21, and 0.86 nm^-1^, respectively. However, the epsilon axis intercept also describes the visible differences. These values are 10.60, 20.40, and 32.57 nm, indicating the scale at which the cluster cores merge and the cluster parameters show a strong epsilon dependence. Note that, for small N values (N<5), there appears to be no N dependence, so these values are not included in the fitting process. This breaking point shows no significant dependence on the nanofocus size, but it could be another quantitative parameter, and a logarithmic representation could also help to highlight how it changes.

| 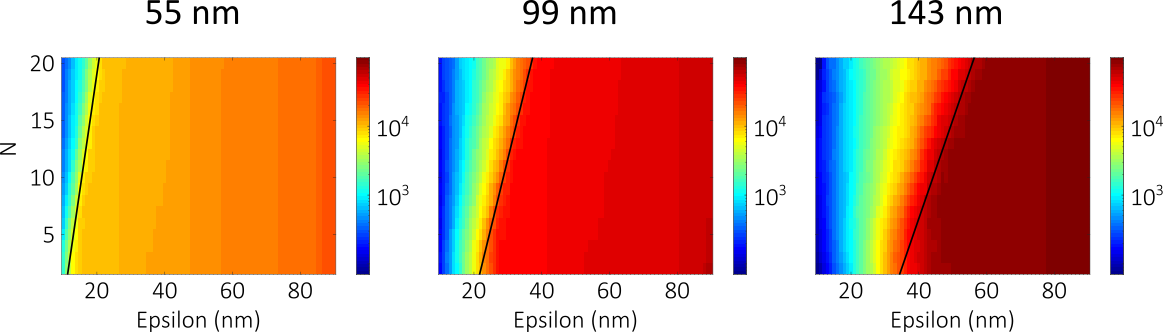 |
| --- |
| *Figure S3: Edge fitting in the cluster area AV maps for different nanofocus sizes (55, 99, and 143 nm)* |

As nonspecific localizations change the maps on the low-N regions, this edge on the maps can characterize the sample. Figure S4 shows the edge fit on the localization number AV maps (for each epsilon, the N position where the derivative of the localization number with respect to N has its maximum value) – note that the third map (nonspecific label density of 50 µm^-3^) is not included in the manuscript. Based on the edge fitting of the previous cluster area maps, only epsilon values above 27 were included in the fitting process. The fitted slopes are 0.004, 0.070, and 0.129 nm^-1^, respectively. These results also show that at higher densities, background localizations have a greater effect at higher values of epsilon.

| 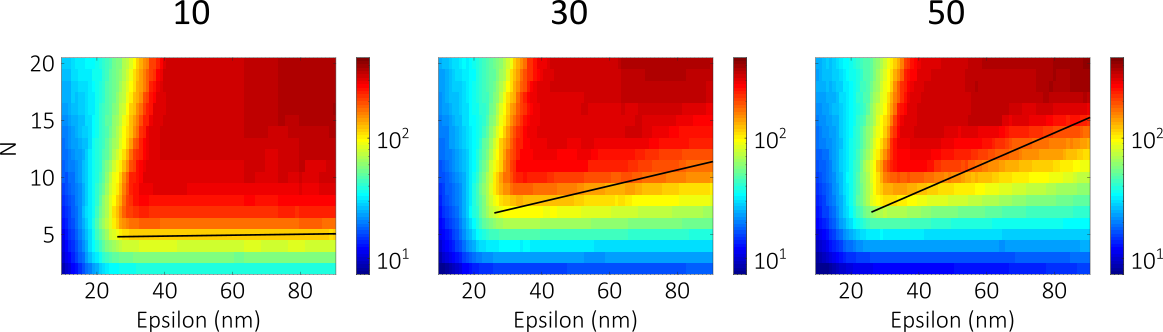 |
| --- |
| *Figure S4: Edge fitting in the localization number AV maps for different nonspecific localization densities (10, 30, and 50 µm^-3^). Note that the map corresponding to the 50 µm^-3^ density is not part of the manuscript* |
